# Serine Biosynthesis with One Carbon Catabolism and the Glycine Cleavage System Represents a Novel Pathway for ATP Generation

**DOI:** 10.1371/journal.pone.0025881

**Published:** 2011-11-02

**Authors:** Alexei Vazquez, Elke K. Markert, Zoltán N. Oltvai

**Affiliations:** 1 Division of Bioinformatics and Surveillance, Department of Radiation Oncology, The Cancer Institute of New Jersey and University of Medicine and Dentistry of New Jersey, Robert-Wood Johnson Medical School, New Brunswick, New Jersey, United States of America; 2 Simons Center for Systems Biology, Institute for Advanced Study, Princeton, New Jersey, United States of America; 3 Department of Pathology, University of Pittsburgh School of Medicine, Pittsburgh, Pennsylvania, United States of America; University of Zaragoza, Spain

## Abstract

Previous experimental evidence indicates that some cancer cells have an alternative glycolysis pathway with net zero ATP production, implying that upregulation of glycolysis in these cells may not be related to the generation of ATP. Here we use a genome-scale model of human cell metabolism to investigate the potential metabolic alterations in cells using net zero ATP glycolysis. We uncover a novel pathway for ATP generation that involves reactions from serine biosynthesis, one-carbon metabolism and the glycine cleavage system, and show that the pathway is transcriptionally upregulated in an inducible murine model of Myc-driven liver tumorigenesis. This pathway has a predicted two-fold higher flux rate in cells using net zero ATP glycolysis than those using standard glycolysis and generates twice as much ATP with significantly lower rate of lactate - but higher rate of alanine secretion. Thus, in cells using the standard - or the net zero ATP glycolysis pathways a significant portion of the glycolysis flux is always associated with ATP generation, and the ratio between the flux rates of the two pathways determines the rate of ATP generation and lactate and alanine secretion during glycolysis.

## Introduction

Oxidative phosphorylation (OxPhos) in the mitochondria is the major pathway for ATP generation in normal cells under normal oxygen conditions (normoxia), generating 32 mole of ATP per mole of glucose [1]. In contrast, under conditions of oxygen limitation (hypoxia), the mitochondrial activity is down-regulated and cells switch to glycolysis for ATP generation that yields only 2 mole of ATP per mole of glucose. Surprisingly, as first observed by Warburg [2], the metabolism of cancer cells is frequently characterized by a significant upregulation of glycolysis even under normoxic conditions, with both an increased glucose uptake and excretion of lactate (Warburg effect, aerobic glycolysis). More recently, it became evident that the Warburg effect is not unique to cancer cells alone. Indeed, both rapidly proliferating normal cells [3], [4], [5], [6], [7], [8] and non-proliferating cells with high metabolic activity [9], [10], [11] display high levels of glycolysis with lactate excretion under normoxic conditions.

Despite the importance of OxPhos and aerobic glycolysis in ATP generation, previous empirical evidence indicates that some cancer cells also utilize an alternative glycolysis pathway with net zero ATP generation [12], [13]. This striking observation implies a physiological role for aerobic glycolysis other than ATP generation. One such role may be the capacity of glycolysis to fulfill the need of rapidly proliferating cells for precursor metabolites. However, it has been shown previously that the need for precursor metabolites in itself is not sufficient to explain the high glycolysis rates observed in proliferating cells [14], [15]. Instead, molecular crowding and its resulting constraint on macromolecular concentrations is the key factor determining the Warburg effect [15], [16]. The high density of macromolecules in the cell imposes limits on the total mitochondrial content per unit of cell volume and the total content of ribosomes and metabolic enzymes as well. In turn, the inherent limitation in mitochondrial density results in an upper bound on the maximum achievable OxPhos capacity. We have shown previously that this maximum is achieved at physiological conditions and that it results in a metabolic switch involving an upregulation of glycolysis and lactate excretion [15], [16]. Yet, all these results were obtained making use of the standard glycolysis pathway, with a yield of 2 moles of ATP per mole of glucose.

Here we investigate the metabolic flux redistributions in proliferating cells that utilize the alternative glycolysis pathway with net zero ATP production [12], [13]. To this end we improve on our previous flux balance model of human cell metabolism [15] by more precisely accounting for protein synthesis, including a self-consistent constraint that all ribosomal-, enzyme associated-, and non-metabolic proteins need to be accounted for by the rate of protein synthesis, which is proportional to the ribosomal density. We also make a more precise accounting of the molecular crowding constraint by considering mitochondria as a subcellular compartment independent from the cytosol. Using this model we uncover a novel pathway for ATP generation that involves reactions in the serine biosynthesis, one-carbon metabolism and the glycine cleavage system, and show that the pathway is transcriptionally upregulated in an inducible murine model of Myc-driven liver tumori-genesis. The flux rate of this pathway is predicted to be two-fold higher in cells with net zero ATP glycolysis relative to cells with the standard glycolysis. Furthermore, it accounts for most of the glycolysis rate in cells with net zero ATP glycolysis.

## Results

### Flux balance model of cell metabolism with molecular crowding constraint

As starting point, we utilize a genome-scale metabolic reconstruction of a generic human cell [17] that includes most biochemical reactions catalyzed by enzymes encoded in the human genome. We add auxiliary reactions to represent nutrient uptake, excretion of metabolic byproducts, basal ATP demand needed for cell maintenance, basal rate of protein degradation, synthesis of cell biomass components (proteins, lipids, RNA and DNA) and cell proliferation (biomass components → cell) (Table S1). We assume that the cell is in a steady state where the production and consumption of every metabolite and macromolecules balances, known as the flux balance constraint [17]. We use *S_mi_* to denote the stoichiometric coefficient of metabolite *m* in reaction *i*. We use *f_i_* to denote the steady state reaction rate (flux) of the *i* ^th^ reaction in the metabolic network, where all reversible reactions are represented by a forward and backward rate, respectively. Reactions are divided into nutrient import reactions (*RI*), reactions taking place outside the mitochondria (*RnM*) and reactions taking place in the mitochondria (*RM*). We use *φ_c_* to denote the relative cell volume fraction occupied by the *c* ^th^ cellular compartment, where a compartment represents the overall contribution of macromolecules of certain type (e.g., ribosomes) or of certain cell organelle (e.g., mitochondria). Specifically, here we consider proteins that do not form part of enzyme complexes or ribosomes (*P0*), all metabolic enzymes catalyzing reactions outside the mitochondria (*EnM*), all metabolic enzymes catalyzing reactions in the mitochondria (*EM*), ribosomes (*R*), and mitochondria (*M*). We assume the proliferation rate (*μ*) and the total relative volume fraction occupied by macromolecules and organelles (*φ_max_*) are known and are given as input parameters of the model. Finally, we estimate the metabolic fluxes and compartment densities as the solution of the following optimization problem:

Find the *f_i_* and *φ_c_* that minimize the sum of nutrient import costs
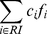
(1)subject to the metabolic constraints: flux balance constraints
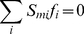
(2)minimum/maximum flux constraints
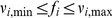
(3)minimum/maximum volume fraction constraints
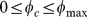
(4)molecular crowding constraints
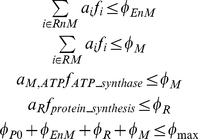
(5)where *c_i_* is the nutrient import cost associated with the uptake reaction *i*, *a_i_* = *v_i_*/*k_eff,i_* are the crowding coefficients of metabolic enzymes (enzyme molar volume/enzyme effective turnover) [18], *a_R_* = *v_R_*/*k_R_* is the ribosome crowding coefficient (ribosome molar volume/protein synthesis rate per ribosome), and *a_M,ATP_* = *v_s,M_*/*r_M_* the crowding coefficient of mitochondria ATP generation (ATP synthesis rate per mitochondria mass/mitochondria specific volume) [15], [16].

The estimation of all the model parameters is presented in the Methods section. Here we discuss some of them that deserve particular attention. The cost of importing molecules is in general determined by their size, charge and hydrophilic properties. In a first approximation we assume that the cost of importing molecules is proportional to their size and use the molecular weight as a surrogate for size. Within this approximation the import cost *c_i_* is equal to the molecular weight of the molecule imported in the auxiliary uptake reaction *i* (Table S1). The effective turnover numbers *k_eff,i_*, quantify the reaction rate per enzyme molecule. For example, for an irreversible single substrate reaction satisfying Michaelis-Menten kinetics, *k_eff_* = *kS*/(*K*+*S*), where *k* is the enzyme turnover number, *K* the half-saturation concentration and *S* the substrate concentration. The turnover numbers of some human enzymes are reported in the BRENDA database [19]. They have a typical value of 10 sec^−1^ and a significant variation from 1 to 100 sec^−1^ (Table S2). However, for most reactions we do not know the turnover number, the kinetic model, or the metabolite concentrations, impeding us to estimate *k_eff_*. To cope with this indeterminacy we performed a sampling strategy, whereby the *k_eff,i_* were sampled from a reasonable range of values, and then focused on the predicted average behavior and 90% confidence intervals (see Methods for additional details). The typical enzyme crowding coefficient is about *a_i_*∼0.00013 (mM/min)^−1^, which is interpreted as follows: to maintain a reaction rate of 1 mM/min we need to allocate a relative cell volume of 0.00013 (0.013%) for the corresponding enzyme. The crowding coefficients are significantly larger for ribosomes and the mitochondria: *a_R_* = 3.6 (mM/min)^−1^ and *a_M,ATP_* = 0.017 (mM/min)^−1^, respectively.

The flux balance equation for proteins (equation (2) with *m* = *proteins*) is formulated more generally than before. Previous models have assumed a constant protein concentration and have not taken into account the self-consistent need to synthesize all the proteins in enzyme complexes and ribosomes [14], [15]. In contrast, here we account for three major categories, proteins not associated with metabolism, proteins that are components of enzyme complexes, and ribosomal proteins, with their concentrations (moles/cell volume) denoted by *P*_0_, *P_E_*, and *P_R_*, respectively. In proliferating cells, these concentrations will decrease at a rate (*μ*+*k_D_*)(*P*_0_+*P_E_*+*P_R_*), where *μ* denotes the proliferation rate and *k_D_* the basal rate of protein turnover. The total concentration of proteins in enzyme complexes can be estimated as *P_E_* = *n_PE_E* = *n_PE_*Σ*_i_f_i_*/*k_eff,i_*, where *n_PE_* is the average number of proteins in an enzyme complex (about 2.4) and *E* is the total concentration of metabolic enzymes. Similarly, *P_R_* = *n_PR_φ_R_*/*v_R_*, where *n_PR_* is the number of proteins in a ribosome (82 for the 80S ribosomes) and *φ_R_*/*v_R_* is the concentration of ribosomes. Putting all these elements together, the balance between protein turnover and synthesis implies *f_Protein_sysnthesis_* = (*μ*+*k_D_*)[*P*_0_+*n_PE_*Σ*_i_*(*f_i_*/*k_eff,i_*)+(*n_PR_*/*v_R_*)*φ_R_*], where the term (*μ*+*k_D_*)*P*_0_ is the only one considered in previous models [14], [15], [17]. In an effective manner, each metabolic reaction contributes to a component of protein synthesis, with a stoichiometric coefficient (*μ*+*k_D_*)*n_PE_*/*k_eff,i_*, quantifying the amount of protein necessary to keep the concentration of the corresponding enzyme constant. Similarly, a constant ribosome volume fraction also accounts for a component of protein synthesis, with a stoichiometric coefficient (*μ*+*k_D_*)*n_PR_*/*v_R_*, quantifying the amount of protein necessary to keep the ribosomes concentration constant. The rate of protein synthesis accounting for this effective protein dilution/degradation thus models the autocatalytic nature of cell metabolism, whereby the macromolecular complexes catalyzing the metabolic reactions ultimately are themselves a product of metabolism.

To model the alternative glycolysis pathway we replaced the pyruvate kinase catalyzed reaction: Phosphoenolpyruvate+ADP+H^+^→Pyruvate+ATP, by the ATP independent reaction: Phosphoenolpyruvate+H^+^→Pyruvate+Phosphate. In the latter reaction phosphoglycerate mutase (PGM1) is a candidate phosphate acceptor, which is then dephosphorylated by a yet unknown mechanism [12].

### Changes in the relative macromolecular densities with increased cell proliferation

**Figure 1:**
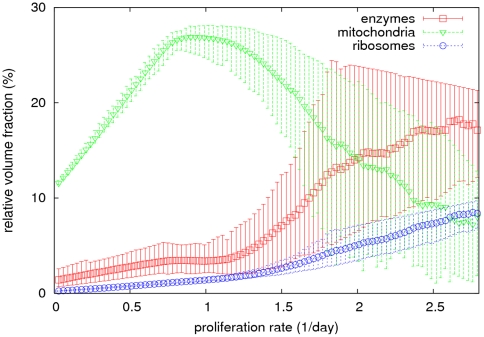
Cell component densities at different proliferation rates. Model predicted relative cell volume fraction occupied by metabolic enzymes (red squares), ribosomes (blue circles) and mitochondria (green triangles), respectively. The model-predicted median and 90% confidence intervals are shown.

Figure 1 shows the predicted relative volume fraction occupied by non-mitochondrial enzymes (*φ_EnM_* = Σ*_i|EnM_a_i_f_i_*), mitochondria (*φ_M_*), and ribosomes (*φ_R_*) as a function of the proliferation rate for cells growing in a medium containing glucose, glutamine, essential amino acids, and oxygen. The ribosomal density increases monotonically with increasing the proliferation rate in a nonlinear fashion, reaching a maximum of 10% of the cell volume at the highest proliferation rate of approximately 2.79/day (minimum doubling time of ln(2)/*μ_max_* = 6 hours) (Fig. 1, blue circles). At low proliferation rates the mitochondrial density increases with increasing the proliferation rate from 10% to about 30% of the cell volume (Fig. 1, green triangles). However, beyond a proliferation rate of about 0.8/day (doubling time 21 hours), the mitochondrial density decreases with increasing the proliferation rate. This is in turn accompanied by a dramatic increase in the density of metabolic enzymes (Fig. 1, red squares). Our model thus predicts that when switching from low to high proliferation rates, the cell makes a transition from a mitochondria dominated molecular crowding regime to one dominated by enzymes + ribosomes (Fig. 1).

The impact of altering the different model parameters on the behavior of the model can also be tested. Larger values of the mitochondrial crowding coefficient *a_M_*, e.g., due to a decrease in mitochondrial efficiency for ATP generation, will cause a decrease of mitochondrial density at lower proliferation rates. Larger ribosome crowding coefficient *a_R_*, e.g., due to a decrease in protein synthesis efficiency, will result in a faster increase of the ribosome density with increasing the proliferation rate, and a consequently faster decrease of the mitochondria density. Similarly, an increase in the average crowding coefficient of metabolic enzymes will cause a faster increase of the total enzyme concentration with increasing the proliferation, resulting in a faster decrease of the mitochondrial density as well. Cancer cells are characterized by partial alterations in all of these components, potentially resulting in a more dramatic effect than that depicted in Figure 1. In particular, mutations leading to damaged mitochondria will enhance the effect, as originally hypothesized by Warburg [2].

### Metabolic switch from low- to high proliferation rates

**Figure 2:**
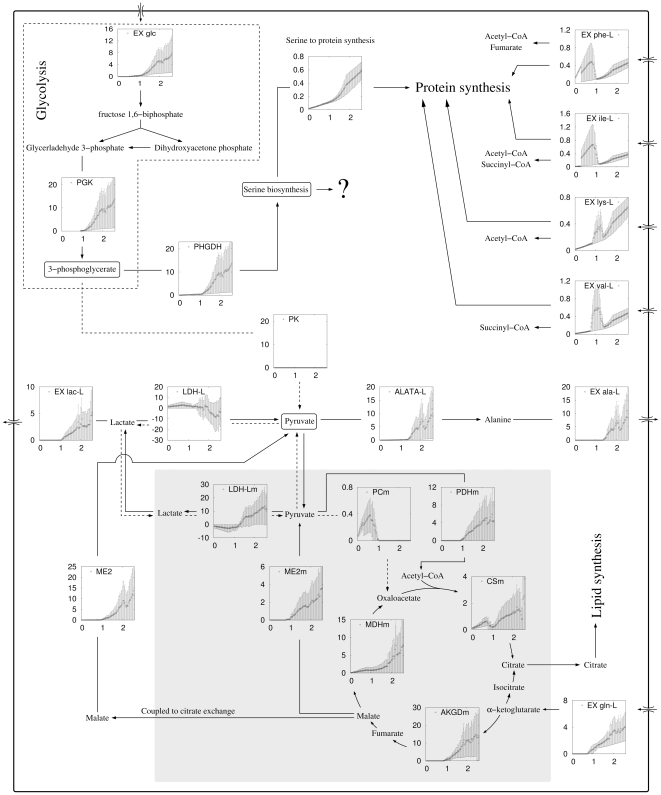
Metabolic switch with increasing proliferation rate. Schematic representation of the flux of selected metabolic reactions and pathways at different proliferation rates, in cells utilizing glycolysis with net zero ATP production. The individual panels show the rate of the indicated reactions (vertical axis, in units of mM/min = mmol/min/L) as a function of the proliferation rate (horizontal axis, in units of 1/day). The gray shadow background contains reactions taking place in the mitochondria. Note, that the predicted metabolic switch is lost if the molecular crowding constraint is removed from the model (see Fig. S1). Abbreviations: metabolite import/export (Ex metabolite: glc = glucose, gln = glutamine, Ala-L = L-alanine, phe-L = L-phenylalanine, ile-L = isoleucine, lys-L = L-lysine, val-L = L-valine, lac-L = L-lactate), phosphoglycerate kinase (PGK), pyruvate kinase (PK), phosphoglycerate dehydrogenase (PGCD), L-alanine transaminase (ALATA-L), malic enzyme (ME), malate dehydrogenase (MDH), pyruvate carboxylase (PC), pyruvate dehydrogenase (PDH), citrate synthase (CS), α-ketoglutarate dehydrogenase (AKGD).

The predicted transition in the macromolecular composition of the cell is accompanied by a global switch in the cell's metabolic state (Fig. 2). At the proliferation rate of about 0.8/day (doubling time 21 hours) the model predicts a substantial increase in glucose uptake, sudden activation of glutamine uptake and α-ketoglutarate dehydrogenase activity, complete deactivation of pyruvate decarboxylase (PCm) and activation of pyruvate dehydrogenase (PDHm). The activity of pyruvate carboxylase in the low proliferation regime, where there is no glutamine uptake, is consistent with recent experimental data showing that pyruvate carboxylase is needed for growth without glutamine [20]. The activation of glutamine uptake at high proliferation rates is also in agreement with what have been observed experimentally [21]. We also observe activation of lactate excretion at high proliferation rates, the hallmark of the Warburg effect.

Several notable changes take place around the pyruvate branching point (Fig. 2). Most noticeably, the glycolysis pathway (Fig. 2) is truncated at 3-phosphoglycerate and the flux over the ATP-decoupled pyruvate kinase-catalyzed reaction is zero at all proliferation rates. We emphasize that we have not imposed a zero flux over this reaction, and the zero flux is a prediction of the model itself. Phenylalanine, isoleucine, lysine and valine are the major sources of TCA cycle intermediates and pyruvate (via malate) at low proliferation rates. However, at high proliferation rates the TCA cycle intermediates and pyruvate are instead generated from glutamine. The cytosolic- (LDH-L) and mitochondrial L-lactate dehydrogenases (LDH-Lm) form a cycle between pyruvate and lactate. At low proliferation rates, LDH-L converts pyruvate to lactate and LDH-Lm converts lactate back to pyruvate, both reactions working at the same rate (Fig. 2, pyruvate-lactate loop, dashed lines). At high proliferation rates the cycle is reverted, LDH-L converting lactate to pyruvate and LDH-Lm pyruvate back to lactate (Fig. 2, pyruvate-lactate loop, solid lines). In the latter case the LDH-Lm catalyzed reaction has a higher rate, resulting in the net production of lactate, which is then excreted. Finally, at high proliferation alanine is produced from pyruvate and is then excreted. We note the amino acid selectivity for pyruvate and TCA cycle intermediates at low proliferation rates depends on the specific choice of nutrient import cost in equation (1). For example, assuming that the cost of nutrient uptake is equal for all nutrients, we obtain that tryptophan is utilized as a source of pyruvate at low proliferation rates (data not shown). However, the high fluxes of glutamine uptake, the 3-phosphoglycerate shift towards serine biosynthesis, and alanine excretion at high proliferation rates, and the results described below are independent of the choice of nutrient cost coefficients.

### Novel pathway for ATP generation

When considering the molecular crowding constraint, our simulations show that at high proliferation rates most of the glycolysis rate is diverted towards the biosynthesis of serine (Fig. 2). However, this flux rate exceeds by more than 10 fold the serine requirements for protein synthesis (Fig. 2, serine to protein synthesis, top center panel). Therefore, we hypothesized that cells utilizing the net zero ATP glycolysis may overexpress some alternative pathway for ATP generation.

**Figure 3 pone-0025881-g003:**
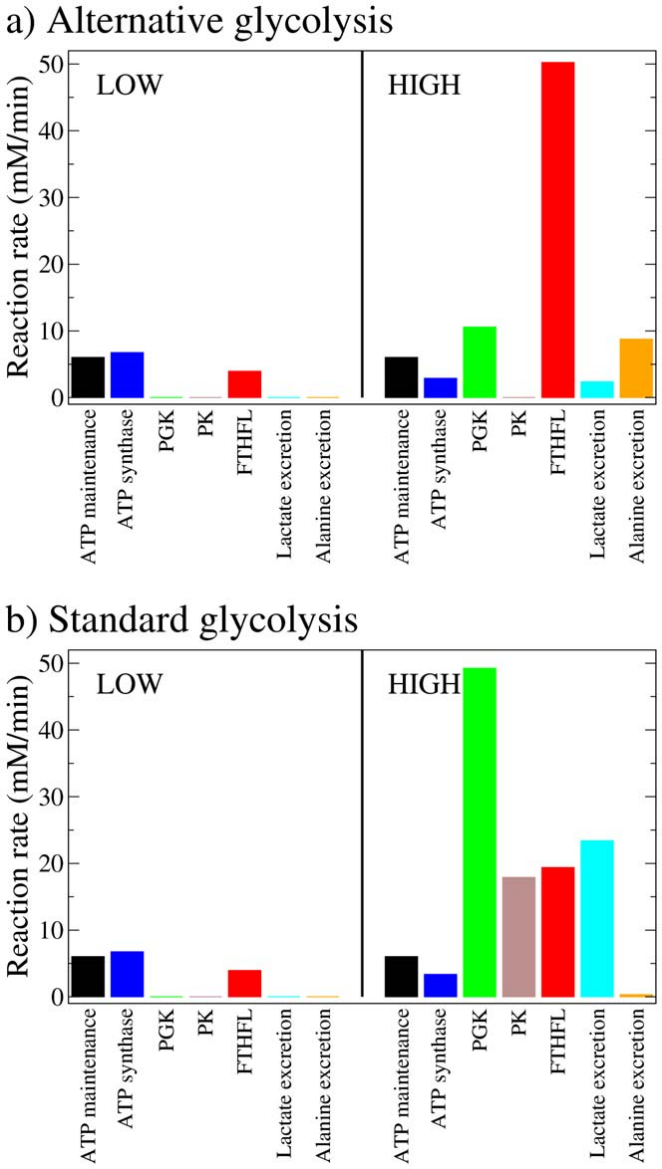
Selected reactions contributing to ATP generation at different proliferation rates. Contribution of ATP synthase, phosphoglycerate kinase (PGK), pyruvate kinase (PK) and formate-tetrahydrofolate ligase (FTHFL) to ATP generation in cells at low (0.03/day, left) and high (2.52/day, right) proliferation rates. The ATP consumed for cell maintenance (black) is shown as a reference. a) cells using the alternative glycolysis pathway with net zero ATP production. b) cells using the standard glycolysis.

To test this hypothesis we inspected the genome-scale reaction rate predictions, focusing on reactions producing ATP. Following this approach we identified the reactions with high rates of ATP production in cells with a net zero ATP glycolysis at different proliferation rates. At low proliferation rates (0.03/day, doubling time 24 days) ATP synthase was the dominant reaction, supplying most of the ATP required for cell maintenance (Fig. 3a,b, left panels). On the other hand, at high proliferations rates (2.79/day, doubling time 6 hours) the formate-tetrahydrofolate ligase (FTHFL), working in the reverse direction to form ATP, is the dominant reaction (Fig. 3a, right panel). Formate-tetrahydrofolate ligase is also active in cells with the standard glycolysis (Fig. 3b, right panel). However, in the case of standard glycolysis phosphoglycerate kinase and pyruvate kinase are the dominant reactions at high proliferation rates (Fig. 3b, right panel). Finally, we note that the molecular crowding constraint is determinant in the differential utilization of ATP synthase and FTHFL at high proliferation rates. After removing this constraint from the model (which mathematically is equivalent to set *φ_max_* = ∞ in equation (5)), we obtain a dramatic increase in the ATP synthase rate and a decrease of the FTHFL rate at high proliferation rates (Fig. S1, HIGH), compared to the predictions with the molecular crowding constraint (Fig. 1a, HIGH).

**Figure 4:**
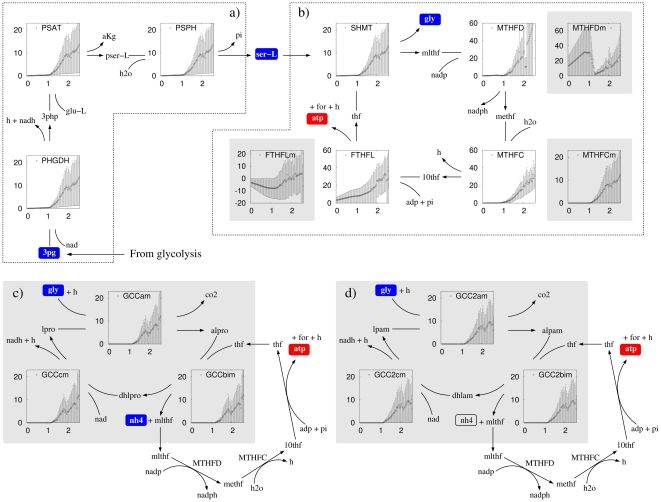
Novel ATP producing pathway. The fate of the high glycolysis flux in cells utilizing the alternative glycolysis [7]. The squared panels show the rate of the indicated reaction (vertical axis, in units of mM/min = mmol/min/L) as a function of the proliferation rate (horizontal axis, in units of 1/day). The gray shadow background contains reactions taking place in the mitochondria. Metabolite and enzyme abbreviations: 3-phosphoglycerate (3 pg), phosphoglycerate dehydrogenase (PHGDH), 3-phosphohydroxypyruvate (3php), L-glutamate (glu-L), phosphoserine transaminase (PSAT), α-ketoglutarate (aKg), L- phosphoserine (pser-L), phosphoserine phosphatase (PSPH), L-serine (ser-L), tetrahydrofolate (thf), serine hydroxymethyltransferase (SHMT), glycine (gly), 5,10-methylene tetrahydrofolate (mlthf), methylenetetrahydrofolate dehydrogenase (MTHFD), 5,10-methenyl-tetrahydrofolate (methf), methenyltetrahydrofolate cyclohydrolase (MTHFC), 10-formyltetrahydrofolate (10thf), formate (for), formate-tetrahydrofolate ligase (FTHFL), S-aminomethyldihydrolipoylprotein (alpro), dyhydrolipolprotein (dhlpro), lipoylprotein (lpro), S-aminomethyldihydrolipoamide (alpam), dihydrolipoamide (dhlam), and lipoamide (lpam). glycine-cleavage complex with lipoylprotein (GCCam, GCCbim and GCCcm) and glycine-cleavage complex with lipoamide (GCC2am, GCC2bim and GCC2cm).

By tracking back the flux from the formate-tetrahydrofolate ligase-catalyzed reaction to glycolysis we uncovered a novel pathway for ATP generation (Fig. 4). The pathway is composed of three main steps. First, *synthesis of L-serine* from the glycolysis intermediate metabolite 3-phosphoglycerate, using NAD and L-glutamate as cofactors (Fig. 4a), with the overall reaction
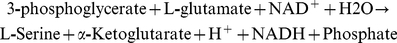
(6)Second, *the conversion of L-serine to glycine* with a concomitant one-carbon metabolism cycle, resulting in the net generation of 1 mole of ATP per mole of serine transformed, using NADP^+^ as a cofactor (Fig. 4b), with the overall reaction
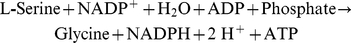
(7)Finally, *the conversion glycine to ammonium (NH_4_)* in the mitochondria with a concomitant one-carbon metabolism cycle, using NAD^+^ and NADP^+^ as cofactors (Fig. 4c or d), with the overall reaction
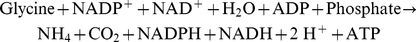
(8)This pathway has a net yield of 2 mole of ATP per mole of 3-phosphoglycerate, therefore 4 mole of ATP per mole of glucose. Furthermore, when compared to the standard glycolysis, the net zero ATP glycolysis manifests a significant decrease in lactate excretion while increasing the alanine excretion (Fig. 3a,b, right panel).

Although the reactions in the reaction cycle shown in Figure 4b are all annotated as reversible in the human metabolic network reconstruction [22], the cycle may not work in the direction of ATP production due to thermodynamic constraints. FTHFL can efficiently catalyze the synthesis of ATP in the bacterium *Clostridium cylindrosporum* [23]. However, it remains to be elucidated whether this is also feasible in human cells, where the tri-functional enzyme C_1_-tetrahydrofolate synthase is responsible for the methylene-tetrahydrofolate dehydrogenase, methenyl-tetrahydrofolate cyclohydrolase and FTHFL activities. To address this issue, we have analyzed a kinetic model of the reaction cycle shown in Figure 4b, focusing on the cytosolic enzymes alone. The model is fully described in the Information S1 and is based on a previous model of folate metabolism [24]. We demonstrate that the kinetic model has a stable steady state in the direction of ATP production, indicating that the novel pathway is thermodynamically feasible.

### The novel ATP-producing pathway is regulated by Myc

**Figure 5:**
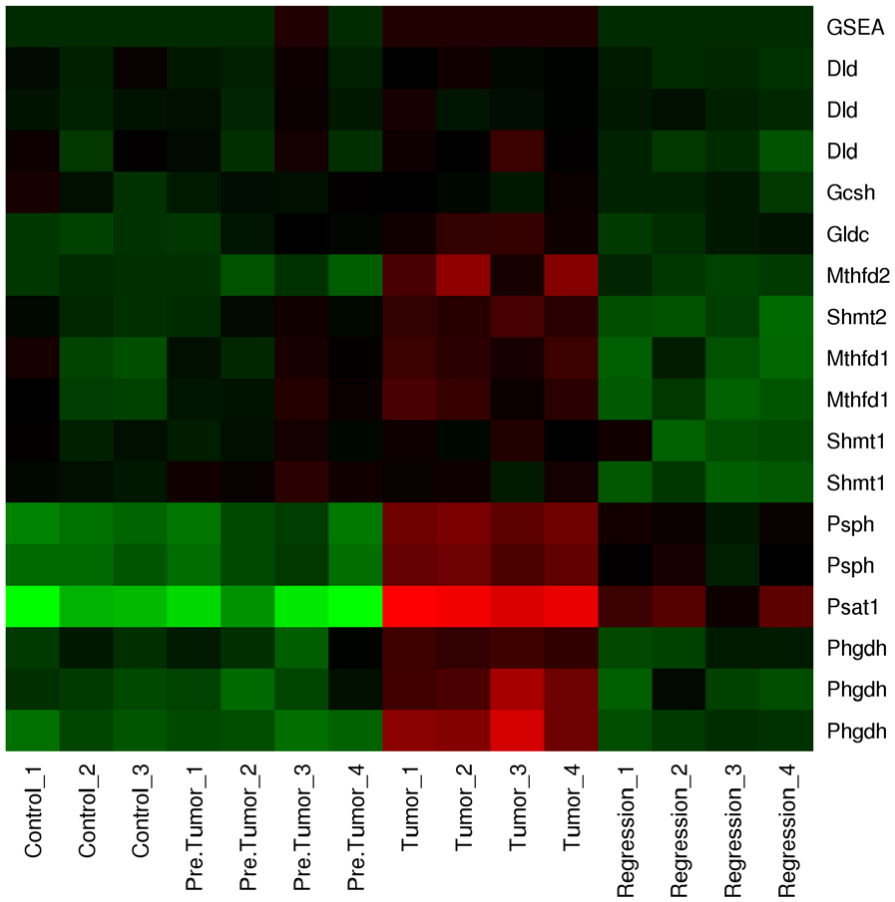
Transcriptional upregulation of the novel pathway in a Myc-induced tumor. Gene expression profiles of genes in the novel ATP producing pathway at different stages of a doxycycline-inducible Myc driven liver cancer [32]. Controls are samples from adjacent normal tissue, “Pre.Tumor” are tumor samples taken 4–5 weeks after Myc induction, “Tumor” are tumor samples taken 8–10 weeks after Myc induction and “Regression” are tumor samples taken 3 days after removal of Myc induction. Color intensity is proportional to change in expression relative to the average across all samples, with red color indicating increased- and green color indicating decreased gene expression. Repeated gene names represent different microarray probes for the same gene.

Consistent with our modeling results, the upregulation of serine and glycine biosynthesis have been observed in some tumor types before [25], [26], [27], [28], [29]. To start to gain insight into this pathway's regulation, we next aimed to identify transcription factors regulating the novel ATP-producing pathway. We performed a search of several transcription factor signatures annotated in the Molecular Signatures Database (MSigDB) [30]. We identified Myc as a transcriptional regulator of six out of the 11 genes in the pathway: *PHGDH*, *PSPH*, *SHMT1*, *MTHFD1*, *MTHFD2* and GCSH (Myc Target Gene Database, [31]), indicating that Myc-induced tumorigenesis may be intimately linked to the activation of this pathway. To test this hypothesis, we have analyzed data from a doxycycline-inducible murine model of Myc-driven liver cancer [32], reporting gene expression microarrays at different tumor stages. We find that the genes coding for the enzymes in the novel ATP-producing pathway are induced following Myc induction, and all, but two (Psat1 and Psph), return to their control levels upon Myc downregulation-induced tumor regression (Fig. 5). Thus, activation of the novel ATP-producing pathway is evident upon Myc-induced tumorigenesis.

## Discussion

The existence of an alternative glycolysis pathway with net zero ATP production in rapidly proliferating cells [12], [13] challenges the general notion that the production of ATP is a major function of glycolysis. Instead, an alternative hypothesis suggests that the increased rate of glycolysis in rapidly proliferating cells is present to support the increased demand for precursor metabolites by anabolic processes involved in cell growth and proliferation [33]. However, based on a partial- or full genome-scale reconstruction of human cell metabolism [17], [22] containing the standard glycolysis pathway, we [15], [16] and others [14] have shown that the anabolic requirements can be satisfied without the need for a dramatic upregulation of glycolysis and the excretion of lactate. We have recapitulated this result here, now using the alternative glycolysis pathway with net zero ATP production, providing *in silico* evidence that the demand for precursor metabolites can be satisfied without upregulation of the alternative glycolysis pathway (Fig. 2).

We have shown previously that molecular crowding is a major determinant of the metabolic changes observed in highly proliferating mammalian- [15], [16] and prokaryotic cells [18], [34] and in quiescent cells with high energy demands [15]. In essence, the high density of macromolecules in the intracellular millieu results in a ‘competition’ among mitochondria, ribosomes, metabolic enzymes and structural protein for the available intracellular space. At low metabolic rates this constraint is less pronounced, and therefore the density of the required organelles and macromolecules can increase to accommodate the increasing metabolic rate. However, just as a finite number of people can be placed in a room, only a finite amount of mitochondria can be present in the cell, resulting in an upper bound for OxPhos capacity. To satisfy its energetic needs beyond this maximum OxPhos capacity the cell need to switch to other pathways that are less costly in terms of the required cell volume fraction to allocate the corresponding enzymes, such as the classic glycolysis pathway. However, this hypothesis has been challenged by the observation that highly proliferating cells utilize an alternative glycolysis pathway with net zero ATP production [12]. To resolve this contradiction we have improved our genome-scale metabolic model of a human cell to be able to investigate the consequences of a net zero ATP production glycolysis.

The results of our *in silico* analyses yield several surprising observations. The glycolysis flux is upregulated in highly proliferating cells and it is routed from 3-phosphoglycerate toward serine biosynthesis. This prediction is supported by experimental observations in both cancer- and highly proliferating normal cells. Rapidly proliferating normal cells (including embryonic cells and adult stem cells) and cancer cells express the M2 isoenzyme of pyruvate kinase (PKM2) [13]. The PKM2 isoform can be present either as a tetramer with high PK catalytic activity or as a dimmer with low catalytic activity, in the latter case greatly reducing the rate over the last steps of standard glycolysis [12], [13], [35]. The upregulation of serine and glycine biosynthesis were observed both in various tumor types [25], [26], [27], [28], [29] and mitogen-activated normal lymphocytes [36], and serum stimulation of Rat1A fibroblast proliferation resulted in an increased ^13^C-labeled glycine derived from 3-phosphoglycerate, in a *myc* dependent manner [37]. Also, when found upregulated, the serine biosynthesis pathway's enzymes are individually essential both for the growth of a subset of breast cancer- and melanoma cell lines [27], [28], [29] and for tumorigenesis in 3-dimensional *in vitro*- [29] and *in vivo* breast cancer models [28]. More importantly, we provided evidence that the expression of genes in this pathway correlates with Myc overexpression in a Myc-driven murine tumor model (Fig. 5). These observations support the activity of the novel ATP-producing pathway in a subset of tumors and, in particular, in Myc driven tumors.

Our *in silico* approach allow us to investigate the fate of the high rate of the serine biosynthesis pathway. We discover that its final endpoint is a novel pathway for ATP generation, starting from the biosynthesis of serine and involving reactions in the one-carbon metabolism pathway and the serine cleavage system (Fig. 4). The reaction responsible for the net ATP generation is catalyzed by formate-tetrahydrofolate ligase (EC 6.3.4.3), working in the ATP production direction. This pathway has a yield of 2 mole of ATP per mole of 3-phosphoglycerate, or 4 moles of ATP per mole of glucose. Taken together our *in silico* evidence indicates that, even in the context of an alternative glycolysis pathway with net zero ATP production, glycolysis is upregulated to satisfy the high energy demand of highly proliferating cells, during conditions where molecular crowding imposes a bound or a reduction in the mitochondrial density.

The novel pathway doubles the ATP yield from 2 to 4 mole of ATP per mole of glucose (Table 1). The novel pathway requires, however, the balance of several co-factors and thus it is coupled to several other reactions. Yet, it remains to be elucidated what the potential evolutionary advantage of having two alternative glycolysis pathways is (i.e., the net zero ATP and the standard pathways). As we show here, the novel pathway can generate two times more ATP, thus an energetic reason is probably likely. In contrast, the novel pathway involves 17 reactions, 7 more than the standard glycolysis, potentially contributing more to molecular crowding. Taken together with OxPhos, we obtain a hierarchy in terms of ATP yield: OxPhos>>net-zero-ATP-glycolysis>standard-glycolysis, and the same hierarchy in terms of molecular crowding. Therefore, these pathways provide the cell with different alternatives to cope with competing efficiency principles, ATP yield per mole of substrate or ATP yield per occupied volume fraction. Concomittantly, other factors, such as the cellular lactate and alanine production also has several potential advantages on the population level that may enhance the invasiveness of tumor cells. Also, tumor cells frequently encounter fluctuating hypoxia levels within growing tumors [38] requiring a capability to rapidly deploy alternative metabolic strategies [39]. In any event, our model identifies several metabolic changes that can be subject to further theoretical and experimental investigations and delineates potential enzyme targets for treatment modalities attempting to interfere with cancer metabolism.

**Table 1:** Overall reaction.

a) Glycolysis
Glucose+2 ADP+2 Phosphate→2 Lactate+**2ATP**+2 H_2_O+2 H^+^
b) Novel ATP generating Pathway
Glucose+2 L-glutamate+6 NAD^+^+4 NADP+6 H_2_O+4 ADP+4 Phosphate→
2 NH_4_+2 a-ketoglutarate+6 NADH+4 NADPH+**4 ATP**+14 H^+^+2 CO_2_

## Materials and Methods

### Metabolic network reconstruction

The reactions annotated in *H. sapiens* metabolic reconstruction 1 were downloaded from the BiGG database [22]. They are listed in Table S1, together with all auxiliary reactions.

### Crowding coefficients

Dividing the mitochondrium specific volume (3.15 mL/g in mammalian liver [40] and 2.6 mL/g in muscle [41]) by the rate of ATP production per mitochondrial mass (0.1–1.0 mmol ATP/min/g [42], [43], [44]) we obtain *a_M_* values between 0.0026 to 0.032 min/mM. Except when specified, we use the median 0.017 min/mM. Dividing the ribosome molar volume (*v_R_* = 4,000 nm^3^×6.02 10^23^/mol = 2.4 L/mmol) by the rate of protein synthesis per ribosome (0.67 proteins/min [45]) we obtain *a_R_* = 3.6 min/mM. The enzyme crowding coefficients were estimated as *a_i_* = *v_E_*/*k_i_*. Multiplying the median molecular weight of human enzymes (98,750 g/mol, BRENDA [19], Table S2) by the enzymes specific volume (approximated by the specific volume of spherical proteins, 0.79 mL/g [46]) we obtain an estimated enzymes molar volume of *v_E_* = 0.078 L/mmol.

### Sensitivity analysis

The turnover numbers of human enzymes *k* have significant variations from 1 to 100 sec^−1^ and the distribution of log_10_(*k*) is approximately uniform in this range (BRENDA, [19], Table S2). Based on this data we sampled the log_10_(*k_eff_*) values from a uniform distribution in the range between log_10_(1) to log_10_(100). At each proliferation rate we run 100 simulations. On each simulation, for each reaction, a value of *k_eff,i_* is extracted from the distribution described above. With this set of *k_eff,i_* parameters we then solve the optimization problem (1)–(5) and obtain estimates for the reaction rates. Based on the 100 simulations we finally estimate the median and 90% confident intervals for the rate of each reaction. This data is reported in Figures 2–4 for selected reactions.

### Macromolecular composition

Proteins were divided into three pools: ribosomal-, components of metabolic enzyme complexes-, and non-metabolic proteins. Each ribosome contributes to *n_PR_* = 82 proteins/ribosome (49 in the 60S and 33 in the 40S subunits [47]). The ribosomal protein concentration was computed as *P_R_* = *n_PR_φ_R_/v_R_*. Each enzyme contributes with *n_PE_* = 2.4 proteins in average, estimated as median enzyme molecular weight (98,750 g/mol, reported above) divided by the median molecular weight of a human protein (40,835 g/mol). The median molecular weight of a human protein was estimated from the median protein length (355 amino acids [48]) and the typical amino acid composition [49]. The enzyme related protein concentration was computed as *P_E_* = Σ*_i_n_PE_f_i_/k_i_*. The concentration of non-metabolic proteins was estimated as 85% (10% metabolic enzymes and 5% ribosomal protein [48]) of the reported total protein content per cell dry weight (0.018 mmol/g DW [49]), i.e. 0.015 mmol/g DW. The lipids, DNA and RNA composition were estimated by their relative abundance in a generic mammalian cell [49]. The abundance per cell dry weight were converted to concentrations after dividing by the typical cell specific volume 4.3 mL/g [50]. This resulted in a concentration of non-metabolic protein of *P*_0_ = 3.59 mM. The maximum macromolecular density of human cells in the absence of osmotic stress is around *φ_max_* = 40% [51].

### Maintenance parameters

The ATP production rate necessary for cell maintenance is 1.55 mmol ATP/g DW/h [49]. The basal protein degradation rate was estimated as *k_D_*(*P*_0_+*P_E_*+*P_R_*), where *k_D_* = 0.01/h [52].

### Simulations

The optimization problem in equations (1)–(5) was solved in Matlab, using the linear programming function linprog. All reversible reactions were represented by an irreversible reaction on each direction with their own effective turnover number *k_eff,i_*. Most flux bounds were set to *v_i,min_* = 0 and *v_i,max_* = ∞, unless specified otherwise (Table S1).

### Kinetic model

The kinetic model is described in the Information S1.

### Microarray data analysis

The Gene expression dataset reported in [32] was downloaded from the Gene Expression Omnibus (GEO:GSE28198)). RMA normalized signals were calculated and mean-centered across samples.

## Supporting Information

Figure S1 **Selected reactions contributing to ATP generation at different proliferation rates.** Contribution of ATP synthase, phosphoglycerate kinase (PGK), pyruvate kinase (PK) and formate-tetrahydrofolate ligase (FTHFL) to ATP generation in cells at low (0.03/day, left) and high (2.52/day, right) proliferation rates, in cells using the alternative glycolysis pathway with net zero ATP production, after removing the molecular crowding constraint. The ATP consumed for cell maintenance (black) is shown as a reference.
(TIF)

Table S1 List of biochemical reactions and auxiliary reactions in the large-scale metabolic model.
(XLS)

Table S2 List of molecular weights and turnover numbers for selected human metabolic enzymes.
(XLS)

Information S1 **Kinetic model of the one carbon metabolism cycle.**
(PDF)
