# Supplementary figures and images for: Serine Biosynthesis with One Carbon Catabolism and the Glycine Cleavage System Represents a Novel Pathway for ATP Generation
Source: PLoS One. 2011 Nov 2;6(11):e25881. doi: 10.1371/journal.pone.0025881 (PMC3206798; doi:10.1371/journal.pone.0025881)

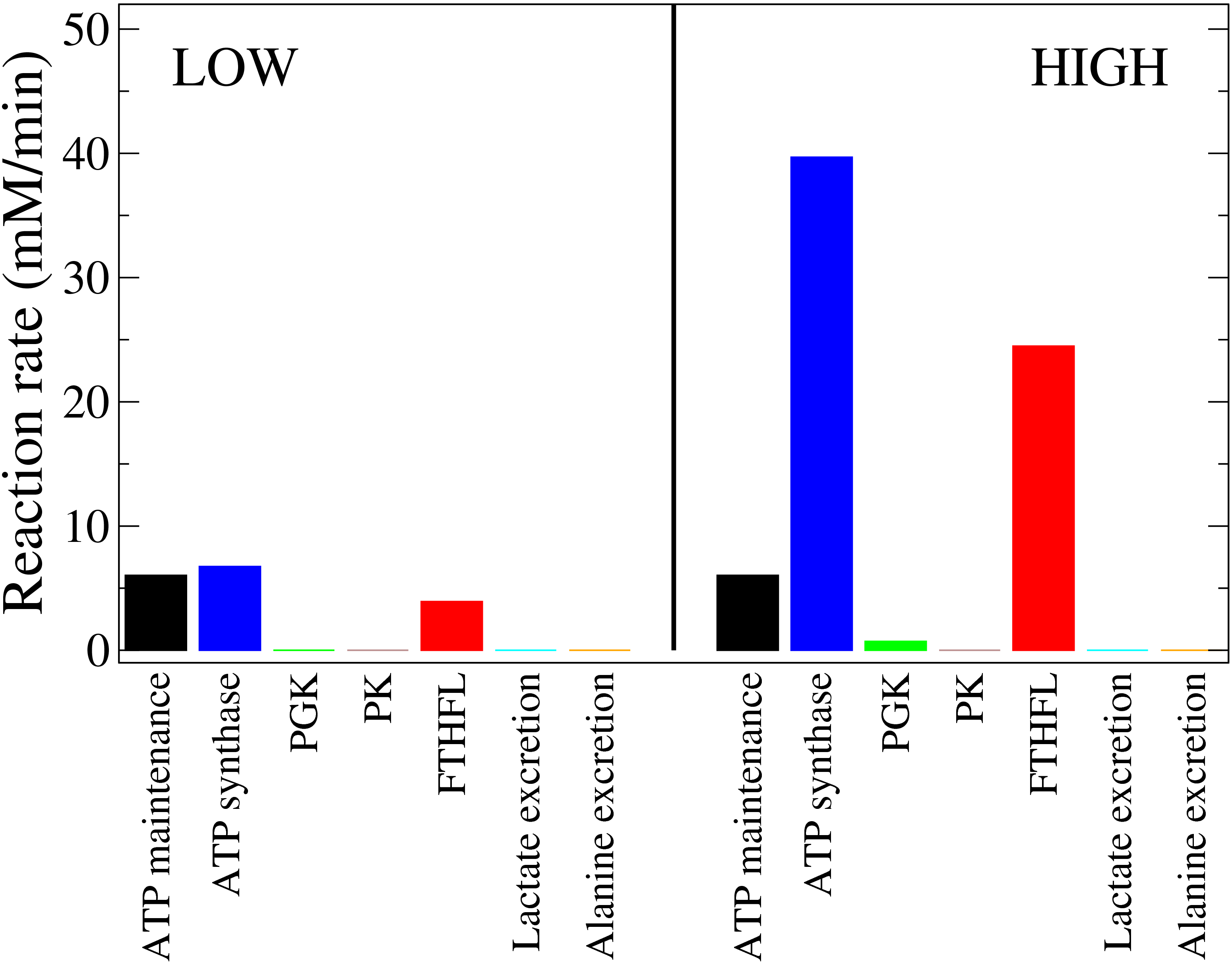

Supplement: Figure S1 — Selected reactions contributing to ATP generation at different proliferation rates. Contribution of ATP synthase, phosphoglycerate kinase (PGK), pyruvate kinase (PK) and formate-tetrahydrofolate ligase (FTHFL) to ATP generation in cells at low (0.03/day, left) and high (2.52/day, right) proliferation rates, in cells using the alternative glycolysis pathway with net zero ATP production, after removing the molecular crowding constraint. The ATP consumed for cell maintenance (black) is shown as a reference. (TIF) [file pone.0025881.s001.tif]
