# Supplementary material for: Serine Biosynthesis with One Carbon Catabolism and the Glycine Cleavage System Represents a Novel Pathway for ATP Generation
Source: PLoS One. 2011 Nov 2;6(11):e25881. doi: 10.1371/journal.pone.0025881 (PMC3206798; doi:10.1371/journal.pone.0025881)
Supplement: Information S1 — Kinetic model of the one carbon metabolism cycle. (PDF) [file pone.0025881.s004.pdf]

Serine biosynthesis with one carbon catabolism and the glycine cleavage system represents a novel pathway for ATP generation
Information S1

Alexei Vazquez[1], Elke K. Markert[2] and Zoltán N. Oltvai[3]
[1] *Department of Radiation Oncology, Bioinformatics & Surveillance,*
The Cancer Institute of New Jersey and University of Medicine and Dentistry of New Jersey,
Robert Wood Johnson Medical School, New Brunswick, NJ, USA
[2] *Simons Center for Systems Biology, Institute for Advanced Study, Princeton, NJ, USA and*
[3] *Department of Pathology, University of Pittsburgh School of Medicine, Pittsburgh, PA, USA*

Kinetic model of the Fig. 3b module

Although the reactions in the reaction cycle shown in Figure 4b of the main text are all annotated as reversible, the cycle may not work in the direction of ATP production due to thermodynamic constraints. To address this issue, we analyze a kinetic model of the reaction cycle shown in Figure 4b, focusing on the cytosolic enzymes alone. The model was based on a previous model of folate metabolism [1]. The four reactions in Fig. 3b are modeled as reversible random multimolecular reactions

$$\begin{aligned} f_{SHMT} &= \left\{ k_{SHMT,f} \frac{[thf]}{K_{SHMT,thf} + [thf]} \frac{[ser-L]}{K_{SHMT,ser-L} + [ser-L]} \right. \\ &- \left. k_{SHMT,r} \frac{[mlthf]}{K_{SHMT,mlthf} + [mlthf]} \frac{[gly]}{K_{SHMT,gly} + [gly]} \right\} E_{SHMT} \end{aligned} \tag{1}$$

$$\begin{aligned} f_{MTHFD} &= \left\{ k_{MTHFD,f} \frac{[mlthf]}{K_{MTHFD,mlthf} + [mlthf]} \frac{[nadp]}{K_{MTHFD,nadp} + [nadp]} \right. \\ &- \left. k_{MTHFD,r} \frac{[methf]}{K_{MTHFD,methf} + [methf]} \frac{[nadph]}{K_{MTHFD,nadph} + [nadph]} \right\} E_{MTHFD1} \end{aligned} \tag{2}$$

$$\begin{aligned} f_{MTHFC} &= \left\{ k_{MTHFC,f} \frac{[methf]}{K_{MTHFC,methf} + [methf]} \right. \\ &- \left. k_{MTHFC,r} \frac{[10fthf]}{K_{MTHFC,10fthf} + [10fthf]} \right\} E_{MTHFD1} \end{aligned} \tag{3}$$

$$\begin{aligned} f_{FTHFL} &= \left\{ k_{FTHFL,f} \frac{[10fthf]}{K_{FTHFL,10fthf} + [10fthf]} \frac{[adp]}{K_{FTHFL,adp} + [adp]} \frac{[pi]}{K_{FTHFL,pi} + [pi]} \right. \\ &- \left. k_{FTHFL,r} \frac{[thf]}{K_{FTHFL,thf} + [thf]} \frac{[atp]}{K_{FTHFL,atp} + [atp]} \frac{[for]}{K_{FTHFL,for} + [for]} \right\} E_{MTHFD1} \end{aligned} \tag{4}$$

where $f_{reaction}$ denotes the net reaction rate, $k_{reaction,f}$ and $k_{reaction,r}$ forward and backward turnover numbers, $K_{reaction,metabolite}$ the half-saturation constant, $[metabolite]$ the corresponding metabolite concentration, and E_{enzyme} the concentration of the corresponding enzyme. Here the following abbreviations have been used: tetrahydrofolate (thf), 5,10-methylene hydrofolate (mlthf), 5,10-methenyltetrahydrofolate (methf), 10-formyltetrahydrofolate (10fthf), L-serine (ser-L), glycine (gly), formate (for), serine hydroxymethyltransferase (SHMT), methylenetetrahydrofolate dehydrogenase (MTHFD), methenyltetrahydrofolate cyclohydrolase (MTHFC), and 5-formethyltetrahydrofolate cycloligase (FTHFL). In humans the cytosolic enzyme activities of MTHFD, MTHFC and FTHFL are carried by the tri-functional enzyme C1-tetrahydrofolate synthase [3], encoded by the MTHFD1 gene. Therefore, the reaction cycle shown in Figure 4b is regulated by the activity of two enzymes, serine hydroxymethyltransferase (SHMT) and C1-tetrahydrofolate synthase (MTHFD1). The total concentration of these two enzymes $E_{SHMT} + E_{MTHFD1}$ determines how fast the system evolves to a steady state and the absolute rate at steady state, but it has no impact on the metabolite concentrations at steady state. Taking this fact into consideration we focus on the system behavior as a function of the relative concentration of one of the enzymes, SHMT for example $E_{SHMT}/(E_{SHMT} + E_{MTHFD1})$.

We focus our analysis on the concentrations of the intermediate metabolites thf, mlthf, methf and 10fthf, assuming the concentration of ser-L, glyc, formate, co-factors and enzymes constant, and given as input parameters of the model (Table S3, below). The concentration of intermediate metabolites evolve in time following the first order differential equations

$$\frac{d[thf]}{dt} = f_{FTHFL}([thf], [10fthf]) - f_{SHMT}([mlthf], [thf]) \tag{5}$$

$$\frac{d[mlthf]}{dt} = f_{SHMT}([mlthf], [thf]) - f_{MTHFC}([mlthf], [methf]) \tag{6}$$

$$\frac{d[methf]}{dt} = f_{MTHFC}([mlthf],[methf]) - f_{MTHFD}([methf],[10fthf]) \tag{7}$$

$$\frac{d[thf]}{dt} = f_{MTHFD}([methf],[10fthf]) - f_{FTHFL}([10fthf],[thf]) \tag{8}$$

Our aim is to determine whether this dynamical system has a steady state in the direction of producing ATP. To this end we numerically determined the fixed point of (5)-(8) that resulted in the highest steady state flux with ATP production. This was accomplished solving the optimization problem:

$$\text{Maximize } f_{FTHFL}([mlthf],[thf]) \tag{9}$$

subject to the stable fixed point constraints

$$\frac{d[thf]}{dt} = \frac{d[mlthf]}{dt} = \frac{d[methf]}{dt} = \frac{d[thf]}{dt} = 0 \tag{10}$$

$$\Lambda \geq 0 \tag{11}$$

and the metabolite concentration bounds

$$0 \leq [thf] \leq [thf]_{max}$$

$$0 \leq [mlthf] \leq [mlthf]_{max}$$

$$0 \leq [methf] \leq [methf]_{max}$$

$$0 \leq [10fthf] \leq [10fthf]_{max} \tag{12}$$

where Λ is the largest eigenvalue of the Jacobian matrix (writing the dynamical system (5)-(8) as $\frac{d\vec{x}}{dt} = \vec{F}(\vec{x})$, the Jacobian matrix is defined as $\frac{\partial F_i}{\partial x_j}(\vec{x})$). The optimization problem was solved in Matlab using the function `fmicon`, with all upper bounds equal to 1 mM, and computing the largest eigenvalue with the Matlab function `eigs`.

For all $0 < E_{SHMT}/(E_{SHMT} + E_{MTHFD1}) < 1$ the steady state with maximum rate is characterized by a positive rate of ATP production (Fig. S2, green circles), indicating that the reaction cycle shown in Figure 4b is thermodynamically feasible in the direction of ATP production. The maximum rate is achieved at a relative concentration $E_{SHMT}/(E_{SHMT} + E_{MTHFD1})$ around 0.05. Hence, the cycle achieves higher rates when the relative concentration of SHMT is much higher than that of MTHFD1. We also note the maximum rate calculated from the specified kinetic parameters is much higher than the median obtained from simulations applying a two-fold change in the model kinetic parameters (Fig. S2, red squares). This could indicate that the kinetic parameters in this pathway have been selected for maximum ATP production. However, since the curve for the observed kinetic parameters is still within the 90% confidence intervals (Fig. S2, red errorbars) we cannot exclude this is just coincidence.

Parameter	Value	Source
SHMT		
$k_{SHMT,f}$	9.58	[3]
$k_{SHMT,r}$	0.60	Estimated as $k_{SHMT,f} V_{max,r}/V_{max,f}$
$V_{max,f}$	40,000	[1]
$V_{max,r}$	25,000	[1]
$K_{SHMT,thf}$	0.05	[1]
$K_{SHMT,ser-L}$	0.6	[1]
$K_{SHMT,mlthf}$	3.2	[1]
$K_{SHMT,gly}$	10	[1]
MTHFD		
$k_{MTHFD,f}$	10	[4]
$k_{MTHFD,r}$		$k_{MTHFD,f} V_{max,r}/V_{max,f}$
$V_{max,f}$	200,000	[1]
$V_{max,r}$	594,000	[1]
$K_{MTHFD,mlthf}$	0.002	[1]
$K_{MTHFD,nadp}$	0.022	[4]
$K_{MTHFD,methf}$	0.01	[1]
$K_{MTHFD,nadph}$	0.022	Estimated as $K_{MTHFD,nadph}$
$[nadp]$	0.02	[5]
$[nadph]$	0.01	[5]
MTHFC		
$k_{MTHFC,f}$	134	[6]
$k_{MTHFC,r}$	3.35	Estimated as $k_{MTHFC,f} V_{max,r}/V_{max,f}$
$V_{max,f}$	800,000	[1]
$V_{max,r}$	20,000	[1]
$K_{MTHFC,methf}$	250	[1]
$K_{MTHFC,10fthf}$	100	[1]
FTHFL		
$k_{FTHFL,f}$	0.23	Estimated from (4) at equilibrium ($f_{FTHFL} = 0$)
$[10fthf]_{eq}$	4	*Clostridium cylindrosporum* [7]
$[adp]_{eq}$	4	*Clostridium cylindrosporum* [7]
$[pi]_{eq}$	4	*Clostridium cylindrosporum* [7]
$[thf]_{eq}$	0.9	*Clostridium cylindrosporum* [7]
$[atp]_{eq}$	0.8	*Clostridium cylindrosporum* [7]
$[for]_{eq}$	2.3	*Clostridium cylindrosporum* [7]
$k_{FTHFL,r}$	0.0364	[8]
$K_{FTHFL,10fthf}$	10	*Clostridium cylindrosporum* [9]
$K_{FTHFL,adp}$	0.0364	[8]
$K_{FTHFL,pi}$	4	[8]
$K_{FTHFL,thf}$	0.364	[8]
$K_{FTHFL,atp}$	0.0302	[8]
$K_{FTHFL,for}$	0.0367	[8]
$[adp]$	0.011	[10]
$[pi]$	6	[10]
$[atp]$	5	[10]
$[for]$	0.9	[1]

Table S3: Model parameters. All half-saturation constants $K_{reaction,metabolite}$ and metabolite concentrations $[metabolite]$ are expressed in mM and all turnover numbers $k_{reaction,direction}$ in 1/sec.

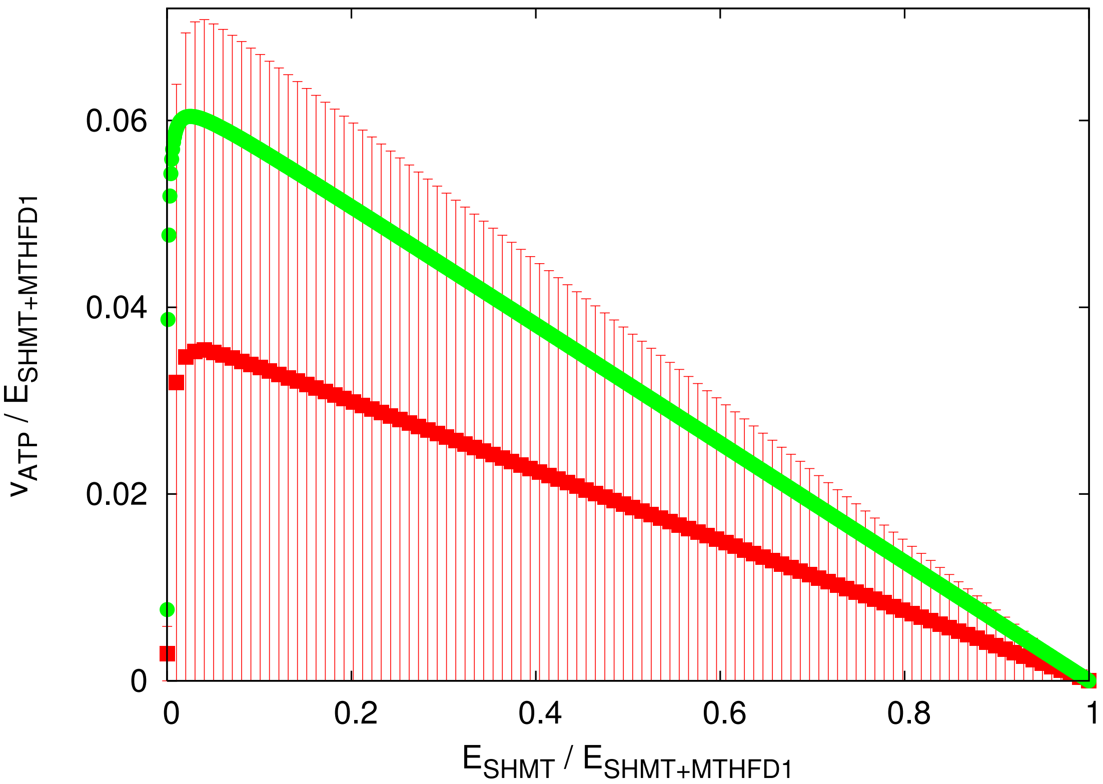

Figure S2: Kinetic properties of the reaction cycle shown in Figure 4b. Maximum ATP production rate of cycle in Figure 4b as a function of $E_{SHMT}/(E_{SHMT}+E_{MTHFD1})$. The green squares were obtained using the kinetic parameters reported in Table S3. The red squares and errorbars represent the median and 90% confidence interval over 100 simulated kinetic parameters, where the logarithm of each kinetic parameter was sampled uniformingly from a value two fold lower to a value two fold higher than the value in Table S3.

[1] Nijhout HF, Reed MC, Budu P, Ulrich CM (2004) A mathematical model of the folate cycle: new insights into folate homeostasis. J Biol Chem 279:55008-16.

[2] Scheer M, Grote A, Chang A, Schomburg I, Munaretto C, Rother M, Söhngen C, Stelzer M, Thiele J, Schomburg D (2011) BRENDA, the enzyme information system in 2011. Nucleic Acids Res 39:D670-6.

[3] Kruschwitz H, Ren S, Di Salvo M, Schirch V (1995) Expression, purification, and characterization of human cytosolic serine hydroxymethyltransferase. Protein Expr Purif 6:411-6.

[4] Pawelek PD, MacKenzie RE (1998) Methenyltetrahydrofolate cyclohydrolase is rate limiting for the enzymatic conversion of 10-formyltetrahydrofolate to 5,10-methylenetetrahydrofolate in bifunctional dehydrogenase-cyclohydrolase enzymes. Biochemistry 37:1109-15.

[5] Pinkas-Sarafova A, Markova NG, Simon M (2005) Dynamic changes in nicotinamide pyridine dinucleotide content in normal human epidermal keratinocytes and their effect on retinoic acid biosynthesis. Biochem Biophys Res Commun 336:554-64.

[6] Pawelek PD, Allaire M, Cygler M, MacKenzie RE (2000) Channeling efficiency in the bifunctional methylenetetrahydrofolate dehydrogenase/cyclohydrolase domain: the effects of site-directed mutagenesis of NADP binding residues. Biochim Biophys Acta 1479:59-68.

[7] Himes RH, Rabinowitz JC (1962) Formyltetrahydrofolate synthetase. II. Characteristics of the enzyme and the enzymic reaction. J Biol Chem 237:2903-14.

[8] Christensen KE, Rohlicek CV, Andelfinger GU, Michaud J, Bigras JL, Richter A, Mackenzie RE, Rozen R (2009) The MTHFD1 p.Arg653Gln variant alters enzyme function and increases risk for congenital heart defects. Hum Mutat :212-20.

[9] Buttlaire DH (1980) Purification and properties of formyltetrahydrofolate synthetase. Methods Enzymol 66:585-99.

[10] Kushmerick MJ, Moerland TS, Wiseman RW (1992) Mammalian skeletal muscle fibers distinguished by contents of phosphocreatine, ATP, and Pi. Proc Natl Acad Sci USA 897521-5.
